# Hepcidin Increases Cytokines in Alzheimer’s Disease and Down’s Syndrome Dementia: Implication of Impaired Iron Homeostasis in Neuroinflammation

**DOI:** 10.3389/fnagi.2021.653591

**Published:** 2021-04-30

**Authors:** Animesh Alexander Raha, Seyedeh Deniz Ghaffari, James Henderson, Subhojit Chakraborty, Kieren Allinson, Robert P. Friedland, Anthony Holland, Shahid H. Zaman, Elizabeta B. Mukaetova-Ladinska, Ruma Raha-Chowdhury

**Affiliations:** ^1^John van Geest Centre for Brain Repair, Department of Clinical Neuroscience, University of Cambridge, Cambridge, United Kingdom; ^2^Clinical Pathology, Addenbrooke’s Hospital, Cambridge University Hospitals NHS Foundation Trust, Cambridge, United Kingdom; ^3^Department of Neurology, University of Louisville, Louisville, KY, United States; ^4^Cambridge Intellectual and Developmental Disabilities Research Group, Department of Psychiatry, University of Cambridge, Cambridge, United Kingdom; ^5^Cambridgeshire and Peterborough Foundation NHS Trust, Cambridge, United Kingdom; ^6^Department of Neuroscience, Psychology and Behaviour, University of Leicester, Leicester, United Kingdom; ^7^The Evington Centre, Leicestershire Partnership NHS Trust, Leicester, United Kingdom

**Keywords:** Alzheimer’s disease, Down’s syndrome dementia, ferritin, hepcidin, choroid plexus, macrophage activation syndrome, neuroinflammation

## Abstract

The liver-derived hormone hepcidin, a member of the defensin family of antimicrobial peptides, plays an important role in host defense and innate immunity due to its broad antibacterial and antiviral properties. Ferritin, an iron storage protein is often associated with iron deficiency, hypoferritinemia, hypoxia, and immune complications, which are all significant concerns for systemic infection in Alzheimer’s disease (AD) and Down’s syndrome (DS) dementia. Serum and post-mortem brain samples were collected from AD, DS and age-matched control subjects. Serum samples were analyzed with ELISA for ferritin, hepcidin and IL-6. Additionally, post-mortem brain sections were assessed by immunohistochemistry for iron-related and inflammatory proteins. A significant increase in serum hepcidin levels was found in DS, compared to controls and AD subjects (*p* < 0.0001). Hepcidin protein was visible in the epithelial cells of choroid plexus, meningeal macrophages and in the astrocytes close to the endothelium of blood vessels. Hepcidin co-localized with IL-6, indicating its anti-inflammatory properties. We found significant correlation between hypoferritinemia and elevated levels of serum hepcidin in AD and DS. Hepcidin can be transported *via* macrophages and the majority of the vesicular hepcidin enters the brain *via* a compromised blood brain barrier (BBB). Our findings provide further insight into the molecular implications of the altered iron metabolism in acute inflammation, and can aid towards the development of preventive strategies and novel treatments in the fight against neuroinflammation.

## Introduction

Dementia is a global public health challenge for this generation, and the most prevalent cause of dementia is late onset of Alzheimer’s disease (LOAD), a fatal neurodegenerative disorder characterized by progressive cognitive and functional impairment associated with memory loss (Hardy et al., 1998). The two primary pathological hallmarks of AD are senile plaques (SP), which are extracellular deposits of Aβ derived from the β-amyloid precursor protein (APP), and neurofibrillary tangles (NFTs), primarily composed of hyper-phosphorylated tau (Goedert et al., 1995; Hardy and Selkoe, 2002). Down’s syndrome (DS) is an aneuploidy due to triplication of all or part of chromosome 21, where the amyloid precursor protein (APP) gene is encoded, and plays a key role in the pathogenesis of AD dementia in DS (Wisniewski et al., 1985; Mann, 1988; Raha et al., 2013). Although dysfunction of APP processing is believed to be the key upstream factor in the pathogenesis of AD (Wilcock, 2012a), neuroinflammation and activation of innate immunity are considered early events in the genesis of AD and in DS dementia (Wilcock, 2012b). In DS and in LOAD, neuroinflammation has been linked to both the exacerbation of SP and NFT pathology, as well as the clearance of Aβ from amyloid plaques (Bell et al., 2007; Mawuenyega et al., 2010; Wildsmith et al., 2013).

The main cell types involved in the brain neuroinflammatory responses are microglia, astrocytes and to a lesser extent, the peripheral and meningeal macrophages (Xue and Streit, 2011; Walsh et al., 2014; Mammana et al., 2018). Microglia in the vicinity of the pathological hallmarks of AD become activated and release a cocktail of inflammatory cytokines and chemokines (Jorda et al., 2020). The systemic infections and its ability to contaminate macrophages, microglia and astrocytes in the central nervous system (CNS) have a particular role (Merad and Martin, 2020). The viruses and bacteria can activate glial cells and induce a pro-inflammatory state in the brain (Kanberg et al., 2020).

Acute infection initiates complex systemic inflammatory responses as part of the innate immunity (Chen et al., 2020). An extensive interaction between the brain and the immune system is present in neurodegenerative diseases (Manson et al., 2020). These are often triggered in a subcellular compartment known as the inflammasome, where IL-6 and IL-1 are the main pathological mediators causing cytokine storm during acute infection (Mehta et al., 2020). These cytokine-mediated multisystem inflammatory responses are common in AD, DS, and other age-related dementia and associated with an increased risk of severe infection (Kox et al., 2020).

Inflammation is often accompanied by abnormal iron homeostasis that leads to systemic hypoferritinemia and low iron levels (Raha et al., 2020). The hypoferritinemia can likely be due, at least in part, to inflammation-driven increases in hepcidin concentrations (Litton and Lim, 2019; Hippchen et al., 2020). How iron homeostasis is maintaining in the whole-body as well as inside the brain is crucial, yet the mechanism remain poorly understood.

Ferritin is an iron storage protein. Excess iron stored, in the form of ferritin protein, is found in many neurodegenerative diseases, including AD (Smith et al., 1998; Raha et al., 2013). The age-associated increase in iron stores in the brain tissue appears to be linked to neuroinflammation (Todorich and Connor, 2004). Aβ and ferritin co-localize within the vascular amyloid deposits in the post-mortem AD brain, with iron accumulation being a ready source of redox generated free radicals that promote neuronal cell death (Smith et al., 1997; Atamna and Boyle, 2006; Raha et al., 2013). The understanding of how iron homeostasis is maintained both at cellular and whole-body level has exponentially increased following the identification of a number of iron related proteins, such as the divalent metal transporter 1 (DMT1), ferroportin (FPN) and hepcidin (McKie et al., 2001; Ganz, 2003; Hentze et al., 2010). The liver-derived hormone hepcidin plays a significant role in host defense and innate immunity due to its broad antibacterial and antiviral properties (Ganz, 2003). Hepcidin regulates systemic iron homeostasis by controlling iron flux into the plasma from the duodenum, as well as through iron recycling macrophages binding to its receptor, the iron exporter FPN (Krause et al., 2000; Pigeon et al., 2001; Nemeth et al., 2004b). Low hepcidin levels cause iron overload, whereas high hepcidin levels cause anemia of inflammation by restricting intestinal iron absorption and macrophage associated iron release (Cheng et al., 2011).

Besides the circulating iron levels, inflammation is another factor regulating hepcidin transcription in hepatocytes. Inflammatory cytokines, mainly in the form of IL-6, are released during inflammation and induce hepcidin expression in the hepatocytes *via* the JAK/STAT3 signaling pathway, leading to phosphorylation of STAT3, its translocation to the nucleus and the hepcidin gene activation (Pietrangelo et al., 2007; Eikelenboom et al., 2012b). The inflammation in AD and DS can therefore be an additional reason for the increase in hepcidin levels, like that seen in the systemic environment.

To evaluate hepcidin and ferritin expression in controls, AD and DS subjects, serum and post-mortem brain samples were analyzed to determine the crosstalk between inflammation and iron dysregulation in normal ageing, AD and DS pathology.

## Materials and Methods

### Ethics and Participants

Ethics plus research and development (R&D) and approvals were granted by the National Research Ethics Committee of the East of England—Norfolk and Cambridgeshire and Peterborough NHS Foundation Trust, respectively. Cambridge Health Authorities Joint Ethics Committee granted ethical approval for the use of human brain tissue and serum samples (Project ref no.: REC:15/WM/0379). Written consents were obtained from controls, adult DS participants and subjects with AD with the capacity to consent. Verbal assent was obtained from participants with AD and DS, who lacked the capacity to provide written assent, and this was provided instead by an appointed consultee, in accordance with the Mental Capacity Act of the UK (2005). Information on older controls (*n* = 50), younger controls (*n* = 50), DS (*n* = 47) and AD (*n* = 50) has already been disclosed in a previous publication (Raha-Chowdhury et al., 2018).

### Assessment of Dementia Status

This was undertaken as described previously using the CAMDEX-DS informant interview and the CAMCOG-DS neuropsychological assessment (Annus et al., 2017).

### Blood and Serum-Collection

Whole blood and serum samples were collected from younger human controls (*n* = 50, aged between 30 and 55 years), and older controls (*n* = 50, aged between 56 and 85 years, with intact cognitive functioning and devoid of neurological and mental health disease), 47 DS cases (aged between 32 and 70 years) and assessed at our Research Centre. A cohort of AD cases (*n* = 50, aged between 56 and 85 years) was provided by collaborator (co-author, RF) for DNA and protein analysis. All blood samples were collected for serum in BD vacutainer SST advance tubes (containing inert gel barrier and clot activator coating). Serum and plasma were separated immediately by centrifugation at 2,465 g for 6 min at 4°C, aliquoted, and stored at –80°C until analysis. Biochemical and hematological profiles including serum iron were analyzed by pathology laboratories at Addenbrooke’s Hospital, Cambridge University NHS Foundation Trust, Cambridge, UK.

### Human Post-Mortem Brain Sections

Human post-mortem brain tissues from controls (mean age 60 ± 15 years), DS (mean age 60 ± 15 years) and AD (mean age 82.0 ± 8.0 years; *n* = 10 in each group) were provided by the Cambridge Brain Bank (Table 1). Cambridge Health Authorities Joint Ethics Committee granted ethical approval for use of human brain tissue and serum samples (Project ref no.: REC:15/WM/0379).

**Table 1:** Characteristics of post-mortem brain samples of Alzheimer’s disease (AD), Down’s syndrome (DS) and age-matched control study population, showing age, gender, post-mortem time delay, Braak stage and cause of death.

Case number	Category	Age	Gender	PM delay (h)	Braak stage	Cause of death
**Down’s syndrome (DS)**
DS1	DS	56	F	6	6	Not known
DS2	DS	76	F	18	6	Septicaemia
DS3	DS	46	F	8	2	Not known
DS4	DS	52	M	24	6	Bronchopneumonia
DS5	DS	64	M	31	6	Not known
DS6	DS	66	F	26	5	Not known
DS7	DS	67	M	8	5/6	Alzheimer’s disease (AD), Cerebrovascular disease (CVD)
DS8	DS	52	F	-	6	AD, Lewy body dementia
DS9	DS	52	M	-	5	Traumatic brain injury (TBI)
DS10	DS	76	F	18	6	Dementia
**Alzheimer’s disease (AD)**
AD1	AD	86	F	86	6	Urinary tract infection/Advanced dementia
AD2	AD	88	M	81	6	Urinary tract infection/Addison’s disease and poor immunity/Vascular and Alzheimer’s dementia
AD3	AD	83	M	46	6	Bowel ischaemia/Hypothyroid/ Hypertension/Alzheimer’s/Atrial fibrillation/Chronic kidney disease/Vascular dementia
AD4	AD	88	M	22.3	5	Pneumonia/Aortic stenosis/Mixed dementia/Left cerebellar hemisphere haemorrhage
AD5	AD	70	M	71	6	Pneumonia/Alzheimer’s disease
AD6	AD	79	F	45.3	6	Cerebrovascular accident/Dementia
AD7	AD	78	M	62	6	Alzheimer’s disease
AD8	AD	89	M	44	5	Alzheimer’s disease
AD9	AD	78	M	24	4	Alzheimer’s disease
AD10	AD	95	M	61	1	Alzheimer’s disease
**Controls**						
C1	Normal	66	M	10.3	5	Cerebrovascular disease/Dementia
C2	Normal	45	F	43.3	0	End stage renal failure/diabetic nephropathy
C3	Normal	54	F	10.3	0	Metastatic myxoid liposacroma/Bronchopneumonia
C4	Normal	52	F	30.3	1	Bronchogenic cancer
C5	Normal	75	F	24	2	Cancer of the ovary
C6	Normal	66	F	29.3	2	Metastatic breast cancer
C7	Normal	83	M	45	0	Not known
C8	Normal	68	M	48	0	Not known
C9	Normal	60	F	60	0	Not known
C10	Normal	66	M	74	0	Not known

### Solid-Phase Enzyme Linked Immunosorbent Assay (ELISA)

The human serum ferritin level was measured with the human ferritin kit ELISA (Abcam, cat number Ab108698) and serum hepcidin (human hepcidin Quantikine ELISA kit, cat number DHP250) and IL-6 (human IL-6 Quantikine ELISA kit, cat number S6050, R&D) according to the manufacturer’s instructions. Briefly, for the detection of hepcidin, samples in 96-wells culture plates were incubated overnight with monoclonal antibody (R&D, MAB83071) or anti-hepcidin capture antibody (1 μg/ml; R&D system, cat number 842127). The following day, the plates were washed three times with washing buffer [0.05% Tween 20 in 0.1 M phosphate buffer saline (PBS) pH7.4] and blocked in blocking solution (1% BSA and 0.05% Tween 20 in 0.1 M PBS) for 2 h at room temperature (RT). After blocking, the plates were washed three times with washing buffer and loaded with 10 μl plasma into 90 μl blocking solution and incubated 4 h at RT. All experiments were performed in quadruplicate unless otherwise specified. A recombinant human protein (R&D, cat number 842129) was diluted in assay buffer in a 2-fold serial dilution and used for the standard curve with a concentration range of 1,000, 500, 250, 125, 62, 31, 15, and 0 pg/ml. After 4 h of incubation, the samples were removed and the plates were washed three times for 5 min with washing buffer before incubation for 2 hs at RT for detection with a biotinylated rabbit polyclonal anti-human hepcidin (Abcam, cat number ab30760) antibody (1 μg/ml) diluted in blocking buffer. After three further washing steps, the plates were incubated with anti-rabbit HRP-conjugated secondary antibody (1:4,000) for 1 h followed by three washes. One-hundred microliter of 1-Step ULTRA tetramethylbenzidine (TMB-ELISA, Thermo Fisher Scientific) was added for ~30 min at RT. Finally, 100 μl of 2 M H_2_SO_4_ was added to quench the reaction. Colorimetric quantification was performed with an Infinite M200 plate reader (Tecan) at 450/540 nm.

### Antibodies

A polyclonal rabbit anti-hepcidin 25 (Abcam ab30760) recognising a 2.8 kDa protein (Raha-Chowdhury et al., 2015), human anti-ferritin light chain (Abcam ab69090), human anti-ferritin heavy chain (Abcam ab65080), Anti-FPN (Abcam ab85370), anti-GFAP (Abcam ab48050), anti-CD68 (Sigma–Aldrich, MAB98073), and monoclonal anti-Iba1 (Thermo Fisher Scientific, MAB M1/70), Polyclonal anti-Iba1(Wako cat number 019-19741), monoclonal anti-IL-6 (Thermo Fisher, cat number M620), monoclonal anti-IL-1β (Thermo Fisher Scientific, cat number ILB1-H67), Anti-β amyloid (Covance cat number SIG 39320), Anti-Phospho-Tau (AT8, Thermo Fisher Scientific, cat number MN1020), was used for IHC. The following secondary antibodies were used: biotinylated goat anti-rabbit-Ig and biotinylated horse anti-mouse (both from Vector Laboratories, 1:250 for IHC); Alexa Fluor 568-labeled donkey anti-mouse-Ig, Alexa Fluor 488-labeled donkey anti-rabbit-Ig, and Alexa Fluor 568-labeled donkey anti-goat-Ig (all from Invitrogen, 1:1,000 for immunofluorescence).

### Perls Staining Methods for Brain Iron

Perls Prussian blue method was followed for staining ferric iron: The ferric iron combines with potassium ferrocyanide to form the insoluble Prussian blue precipitate as follows:

$${\text{4FeCl}}_{\text{3 }}\text{(ferrric iron) + }{\text{3K}}_{\text{4}}{\text{Fe(CN)}}_{\text{6}}\text{(potassium ferrocyanide) = }{\text{Fe}}_{\text{4}}{{\text{[Fe(CN)}}_{\text{6}}\text{]}}_{\text{3}}\text{+12KCl (ferric ferrocyanide)}$$

Paraffin fixed brain sections (*n* = 6, in glass slides) were deparaffinized and hydrated with iron free distilled water. The sections were then transferred to a mixture of equal parts of 2% potassium ferrocyanide for Perls staining at pH6 and 2% HCL in distilled water for 20–30 min. The brain sections were then washed in distilled water. All sections were counterstained in eosin, dehydrated, and mounted with a synthetic resin medium (Meguro et al., 2005).

### Congo Red Staining for Amyloid Plaques

Congo red stain applied to the tissue gives the amyloid protein a salmon-pink color and when placed under polarized light, the amyloid proteins have an apple-green birefringence. This apple-green birefringence is considered pathognomonic for amyloid fibril deposits (Howie et al., 2008). Congo red was purchased from Sigma–Aldrich (cat number C6277). It is an organic compound, a sodium salt of 3,3′-[(1,1′-biphenyl)-4,4′-diyl]bis(4-aminonaphthalene-1-sulfonic acid) and is an azo dye. Congo red is water-soluble, yielding a red colloidal solution. Paraffin fixed human brain sections (*n* = 6, in glass slides) were deparaffinized and hydrated with iron free distilled water. The brain sections were then immersed in Congo red dye for 10 min and rinsed thereafter in distilled water. The sections were differentiated quickly (5–10 dips) in alkaline alcohol solution and rinsed in tap water. Slides were counterstained in Gill’s hematoxylin for 30 s and mounted with a synthetic resin medium.

### Immunohistochemistry (IHC)

Paraformaldehyde (PFA) fixed tissues were first quenched with 5% hydrogen peroxide and 20% methanol in 0.01 M PBS for 30 min at RT followed by three rinses for 10 min in 0.01 M phosphate buffer saline (PBS). Non-specific binding sites were blocked using blocking buffer (0.1 M PBS, 0.3% Triton-X 100, and 10% normal goat serum for polyclonal antibodies or 10% normal horse serum for monoclonal antibodies) for 1 h at RT. Tissue sections were incubated overnight with the primary antibody diluted in blocking buffer. Binding of the primary antibody was detected using a biotinylated secondary antibody followed by an avidin-biotin complex conjugated to peroxidase (Elite standard kit SK6100, Vector Laboratories) and DAB substrate (ABC substrate SK-4200, Vector Laboratories).

### Immunofluorescence (IF)

Brain and other sections were blocked using blocking buffer (0.1 M PBS, 0.3% Triton X 100, 10% normal donkey serum) for 1 h at RT, then incubated overnight at 4°C with primary antibody diluted in blocking buffer. Alexa Fluor-conjugated secondary antibodies were used for detection and samples counterstained with 4′6-diamidino-2-phenylindole (DAPI, Sigma). The sections were then mounted on glass slides with coverslips using Fluoro Save (Calbiochem).

### Microscopy

Bright field images were taken and quantified using Lucia imaging software and a Leica FW4000 upright microscope equipped with a SPOT digital camera. Fluorescence images were obtained using a Leica DM6000 wide field fluorescence microscope equipped with a Leica FX350 camera with 20× and 40× objectives. Images were taken through several z-sections and de-convolved using Leica software. A Leica TCS SP2 confocal laser-scanning microscope was used with 40× and 63× objectives to acquire high-resolution images.

### Image and Statistical Analysis

Data were analyzed by paired Student’s *t*-test (two-tailed) for two group comparison, or by ANOVA test for multiple comparison testing. Values in the figures are expressed as mean ± SEM. A one–way ANOVA was used for comparison of data among control, AD and DS and conducted with IBM-SPSS statistic 19 software. Significance was analyzed using GraphPad and *p*-values ≤0.001 were considered significant and are indicated in the corresponding figures and figure legends.

## Results

### Significant Elevation of Serum Hepcidin in DS Subjects

We investigated serum iron, ferritin and hepcidin levels in AD, DS and age-matched control subjects with sandwich ELISA. Serum iron was measured by clinical biochemistry, at the Addenbrooke’s Hospital of Cambridge University NHS Foundation Trust located in Cambridge, UK. All samples were analyzed on the same day, using same standardized protocol to reduce the day-to-day variation.

**Figure 1:**
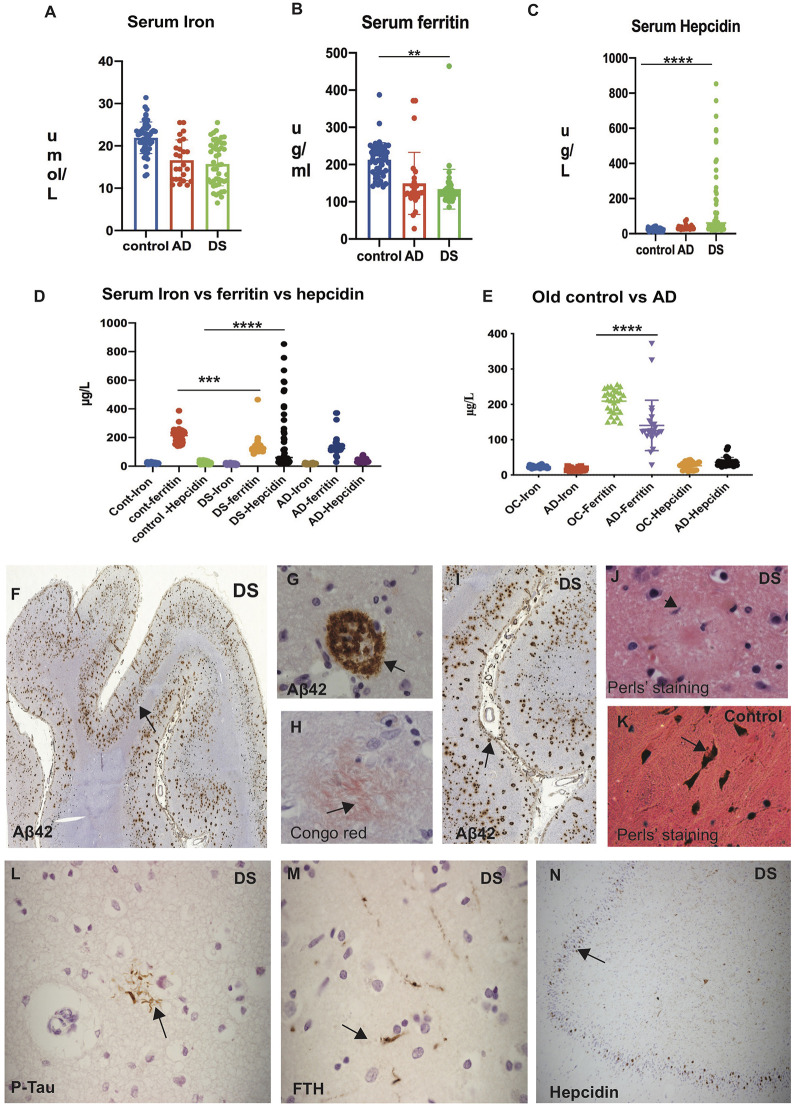
Significant elevation of serum hepcidin in Down’s syndrome (DS) subjects and iron accumulation visible in DS senile plaques. Human serum from Alzheimer’s disease (AD), DS and age-matched control subjects showing iron **(A)**, ferritin **(B)**, and hepcidin (**C**; “Materials and Methods” section described in main text). Scattered plot showing levels of serum iron **(A)** and serum ferritin **(B)** significantly higher in control subjects compared to AD and DS (*p* < 0.0001). In contrast, hepcidin levels were substantially higher in DS compared to AD and controls (*p* < 0.0001; **C**). Comparison of iron, ferritin and hepcidin level showing highest hepcidin levels in DS when compared to controls and AD individuals **(D)**. Serum samples from AD and age matched older controls (OC) on comparison showed the highest ferritin levels in OC compared to AD, whereas there were no significant differences in hepcidin levels **(E)**. Statistical differences were calculated by Mann–Whitney *U* test. **p < 0.001, ***p < 0.0001 and ****p < 0.00001. DS brain sections from superior frontal gyrus (SFG) stained with DAB and anti-Aβ42 antibody revealed strong signal of SPs positive for Aβ42 and visible throughout the cortex (**F**, black arrows highlighting the selected area of plaques formation, **(G)** in higher magnification inside the plaque formation and **(I)** close to the blood vessels. To confirm neurofibrillary (neuritic) plaque formation, a representative section was stained with Congo red **(H)** showing the amyloid proteins having apple-green birefringence under polarized light in senile plaques (SP). Perls’ stain identifies the ferric (Fe3+) form of iron, showing limited ferric iron was visible albeit only in the glial cells periphery of senile plaques **(J)**, and in controls particularly seen in neuromelanin cells of substantia nigra pars compacta (SNPC) in controls **(K)**. Similarly, a section from mid temporal gyrus (MTG) was stained with phospho-tau (AT8) showing neurofibrillary tangle close to glial cells **(L)**, and some of those neuro-filaments were ferritin (FTH) positive **(M)**. In the hippocampus of DS brain section, granule cells were positive for hepcidin **(N)**. Scale bar: **(F** and **I)** = 200 μm, **(L–N)** = 50 μm, **(G,H,J,K)** = 25 μm.

Serum iron levels were significantly higher (*p* < 0.0001) in control subjects (25.07 ± 5.06 μmol/L) compared to AD (16.2 ± 9.2 μmol/L) and DS (15.42 ± 10.1 μmol/L; Table 2, Figure 1A). Similarly, serum ferritin levels were significantly higher (*p* < 0.0001) in controls (216.9 ± 171.1 μg/L), compared to AD (149.4 ± 199.5 μg/L) and DS (133.82 ± 85.7 μg/L; Figure 1B). In contrast, hepcidin levels were substantially higher (*p* < 0.0001) in DS (mean ± SD: 188.32 ± 430.5 μg/L) compared to AD (36.3 ± 18.2 μg/L) and controls (25.38 ± 22.1 μg/L; Figure 1C). The iron and ferritin level in control subjects indicated that iron and ferritin stored were within normal range as expected (Figure 1D). To evaluate that ferritin and hepcidin levels were not reflecting the age, we analyzed age matched controls (ages between 65–85 years) and AD subjects. We found that serum ferritin was significantly higher in aged controls compared to AD (*p* < 0.0001), whereas hepcidin levels were lower in AD (Figure 1E). However, hepcidin levels were high in DS compared to controls and AD individuals suggesting inflammatory changes or impaired dis-erythropoiesis affecting the DS subjects (Figures 1D,E).

**Table 2:** Characteristics of AD, DS and age matched control study population used to measure serum iron, ferritin, hepcidin, and IL-6.

Characteristic	Controls *N* = 50	AD *N* = 50	DS *N* = 47	Significant *p*-value
Males, *n* (%) 25 (50%)	25 (50%)	30 (60%)	25 (53%)	
Females *n* (%)	25 (50%)	20 (4%)	22 (47%)	
Age	50 ± 27	72 ± 23	47 ± 22	
Older controls	*N* = 50, 65± 20			
**Iron parameters**				
Serum iron (μmol/L)	20.18 ± 5.1	15.4 ± 10.1	16.6 ± 9.9	
Serum Ferritin (μg/L)	212.67 ± 72.6	140.5 ± 231.1	133.82 ± 331.5	<0.0001
Serum Hepcidin (μg/L)	25.35 ± 26.6	36.3 ± 51.2	188.32 ± 430.9	<0.0001
Serum IL-6 (pg/ml)	66.23 ± 70.5	378.51 ± 201.89	304.03 ± 330	<0.0001

*Data are expressed as number of patients (percent), mean ± SD. Probability value (p) denote difference among control, AD and DS patient groups. One-way analysis of variance (ANOVA) was used to compare age between groups and statistical analysis was measured with Mann–Whitney *U* test*.

### Iron Accumulation Is Visible in DS Senile Plaques

DS brain sections from superior frontal gyrus (SFG), mid temporal gyrus (MTG), and hippocampus (HP) were then analyzed by immunohistochemistry (IHC) using antibodies specific to Aβ42 (6E10), Phospho-tau (AT8), hepcidin and ferritin heavy chain (FTH) using DAB stain. In the SFG from DS brain, abundant Aβ42 positive plaques were observed in the neocortex (Figures 1F,G, black arrows highlight the selected area of plaques formed) and very close to and even inside the blood vessels (Figure 1I). To confirm neurofibrillary (neuritic) plaque formation, a representative section from DS brain was stained with Congo red (Figure 1H). Congo red stain recognized the amyloid protein appearing as salmon-pink color and when placed under polarized light, the amyloid proteins had an apple-green birefringence. This apple-green birefringence is considered pathognomonic for amyloid fibril deposits (Figure 1H). Similarly, a section from MTG was stained with phospho-tau (AT8) showing neurofibrillary tangle (NFT), close to glial cells (Figure 1L), and some of those neuro-filaments were ferritin (FTH) positive (Figure 1M). In the DS hippocampus, some granule cells were positive for hepcidin (Figure 1N).

To confirm if iron accumulation in the brain tissues were stained with Perls stain to identify ferric form (Fe3+), we followed the procedures described in previous publications (Morris et al., 1992; Meguro et al., 2007), and compared the iron accumulation in aged normal and DS subjects (brain sections from cortex and substantia nigra pars compacta (SNPC) stained with Perls stain). In DS brain, presence of very faint ferric iron was seen around the SP (Figure 1J), whereas strong iron accumulation was seen in the SNPC (in neuromelanin cells; Figure 1K) as reported before (Morris et al., 1992; Meguro et al., 2007).

### Hepcidin Protein Accumulates Around the Senile Plaques in AD and DS Brains

**Figure 2:**
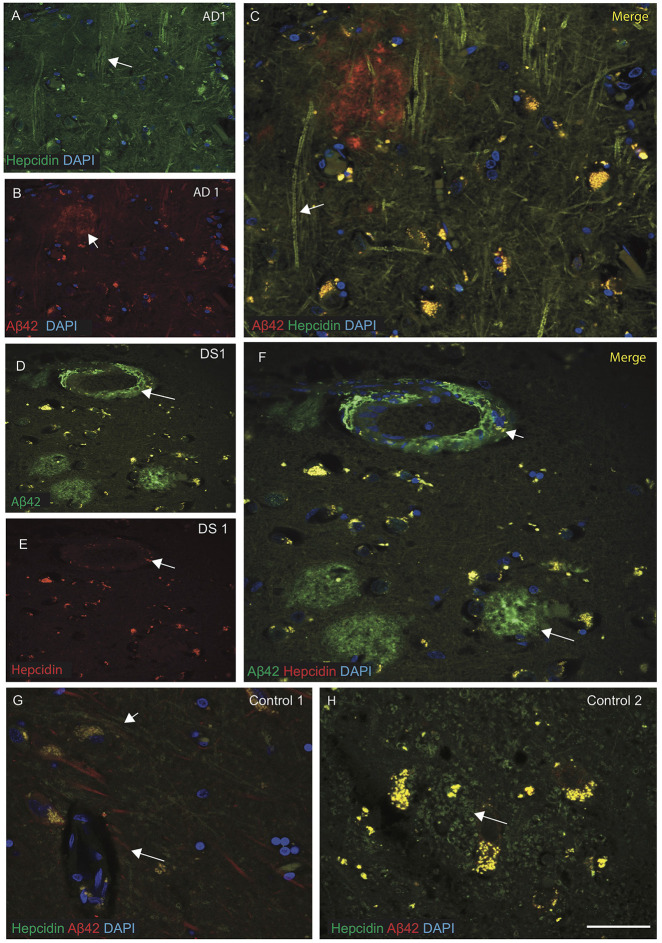
Hepcidin protein accumulates around the senile plaques in AD and DS brains. AD and DS brain sections from the SFG were labeled with double immunofluorescence using anti-Aβ42, anti-hepcidin and counterstained with 4′6-diamidino-2-phenylindole (DAPI) for nuclei (blue) and imaged with confocal microscopy. In AD brain, hepcidin staining was visible in the neuropil and fibrillary structures close to the Aβ42 positive senile plaques (SP) **(A–C)**, white arrows highlighting the selected areas with hepcidin in neuropil and Aβ42 staining in the SP). In DS brain, abundant Aβ42 positive cotton wool appearance of SP, close to the blood vessels were observed, while very limited hepcidin positive glial cells were present in the periphery of plaques **(D–F)**. In contrast, control brains showed co-localization of Aβ42 and hepcidin near the blood vessels **(G)**, while hepcidin predominated in the astrocytes located in the periphery of the glial cells **(H)**. Scale bar: **(A–H)** = 25 μm.

Brain sections from the hippocampus, entorhinal cortex and SFG were then analyzed by IF and imaged with confocal microscopy, using antibodies specific to Aβ42 (6E10) and hepcidin. Sections of AD SFG when stained for hepcidin (green) and Aβ42 (red), showed senile plaques recognized by Aβ42 and hepcidin staining was seen in the neuropil and fibrillary structures (Figures 2A–C). In DS brain, abundant Aβ42 positive cotton wool appearance of senile plaques (SP), close to the blood vessels were observed. In DS SFG, very limited hepcidin positive glial cells were seen in the periphery of plaques (Figures 2D–F). Contrastingly, in control brains (i.e., SFG), abundant hepcidin positive cells were present (Figures 2G,H) wherein the two proteins Aβ42 and hepcidin showed co-localization near the blood vessels, while hepcidin filaments were predominantly located in the astrocytes (Figure 2H).

### Hepcidin and Ferritin Expression in Astrocytes Close to the Blood Vessels

**Figure 3:**
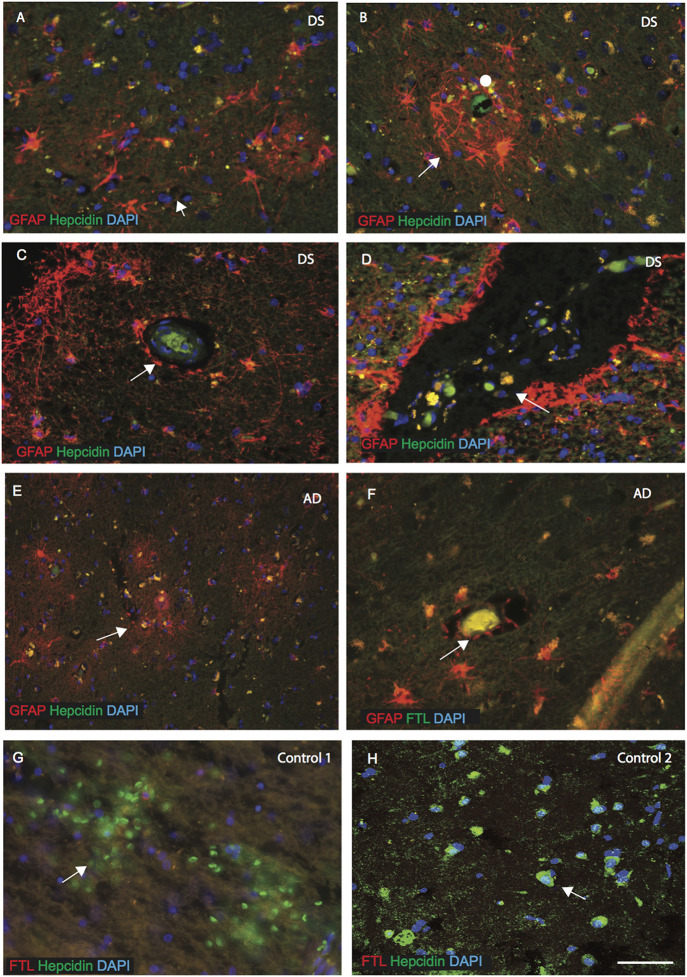
Hepcidin and ferritin expression in astrocytes close to the blood vessels. AD and DS brain sections from the MTG, sub ventricular zone (SVZ) and close to the blood vessels of cortex (SFG) were labeled with double immunofluorescence using anti-hepcidin (green), anti-GFAP or ferritin light chain (FTL; red) and counterstained with DAPI for nuclei (blue) and imaged with confocal microscopy. In DS brain, GFAP positive activated astrocytes were present around the SP and surrounding the blood vessels (**A–B**, indicated with an arrow). Hepcidin expression was visible in the periphery of blood vessels (in the endothelial cells) **(C)**, and co-localized with the GFAP positive astrocytes in the DS and AD brains (**D,E,F**). In the DS brain, particularly in the SVZ, large numbers of small vesicles carrying hepcidin were present and co-localized with GFAP positive astrocytes **(D)**, and near the blood vessels in AD brain **(E)**. The end-feets of astrocytes were surrounding the blood vessels and co-localization with the FTL (green) was visible in the blood vessels **(F)**. In controls, brain section from SFG when labeled with hepcidin and FTL, both proteins were visible in different cells close to the blood vessels, hepcidin most probably in the red blood cells and FTL in the macrophages/microglia **(G)**. In contrast, control brain (in the hippocampus) showed hepcidin to be present in the pyramidal neurons, showing limited co-localization with FTL **(H)**. Scale bar: **(A–H)** = 25 μm.

As described above, hepcidin was observed in the astrocytes in the disease affected brains. We, therefore, evaluated the expression of hepcidin in sections from the MTG, sub ventricular zone (SVZ) and close to the blood vessels of cortex (SFG). In DS brain, GFAP positive activated astrocytes were present around the SP and surrounding the blood vessels (Figures 3A,B, indicated with an arrow or filled circle).

Hepcidin expression was visible in the periphery of blood vessels (in the endothelial cells) and co-localized with the GFAP positive astrocytes in the DS and AD brains (Figures 3C,E,F). In the DS and AD brain, particularly in the SVZ and near the blood vessels, a large number of small vesicles carrying hepcidin were present and co-localized with GFAP positive astrocytes (Figures 3D,E). Similarly, in the AD brain, astrocytes were present surrounding the blood vessels and co-localized with the ferritin light chain (FTL; Figure 3F). Furthermore, from the SFG section of controls, it appeared that soluble hepcidin entering from blood vessels may have been delivered *via* the red blood cells (Figure 3G), whereas ferritin (FTL) may have entered through the macrophages/microglia but did not co-localize with hepcidin close to the blood vessels (Figure 3G). In contrast, hepcidin was present in the pyramidal neurons in the hippocampus of a normal control brain and showed limited co-localization with FTL in the periphery of the neurons (Figure 3H).

### Selective Population of Microglia and Meningeal Macrophages Are Involved in Brain Iron Homeostasis in DS

**Figure 4:**
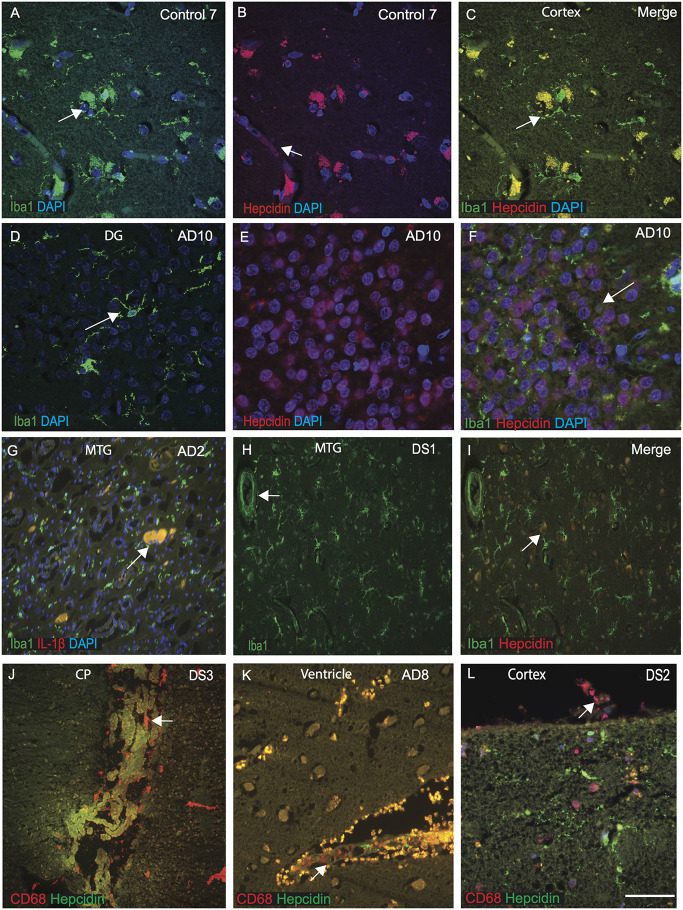
Selective population of microglia and meningeal macrophages are involved in brain iron homeostasis in DS. The brain sections from controls, AD, and DS subjects were labeled with hepcidin and microglial marker (Iba1) or macrophages marker CD68, and counterstained with DAPI for nuclei (blue). Hepcidin was seen in the vesicles, most probably entering from blood vessels and IbaI positive ramified microglia, colocalized in the control cortex **(A–C)**. In AD brain dentate gyrus (DG), showing IbaI positive activated microglia **(D)**, whereas hepcidin was present in the DG granule cells not in the damaged microglia **(E)** with no visible co-localization **(F)**. Iba1 positive microglia may be involved in pruning and clearance of damage cells, where hepcidin protect the granule cells from further damage **(F)**. Brain section from AD (MTG) stained with Iba1 and pro-inflammatory marker IL-1β showing both proteins to be co-localized in the small plaques and activated microglia **(G)**. Cellular damage in this brain section was substantial **(G)**. Similarly, in DS brain section from MTG, IbaI positive activated microglias being present close to the blood vessels **(H)**, but without any co-localization with hepcidin **(I)**. In DS brain, hepcidin protein was visible in the lateral ventricles in a monolayer of epithelial cells of the choroid plexus (CP) and CD68 was found in the macrophages close to epithelial cells of CP suggestive of involvement in transport of serum proteins from macrophages to the brain parenchyma **(J)**. AD brain tissue sections when stained with CD68 and hepcidin show a large number of CD68 positive peri-vascularmacrophages visible around the lateral wall of the 4th ventricles co-localizing with hepcidin at the endothelial margin **(K)**. Similarly, a cortical section from DS labeled with CD68 and hepcidin, show granular hepcidin protein visible in the glial cells inside the cortical layers I and II and CD68 visible in the meningeal macrophages in the meninges **(L)**. These finding support the notion that soluble and vesicular hepcidin could be transport *via* macrophages and blood vessels into the brain parenchyma **(J–L)**. Scale bar: **(A–I)** = 50 μm, **(J–K)** = 70 μm, **(L)** = 20 μm.

Hepcidin and ferritin could be involved in microglial activity and in the amyloid clearance process (Raha et al., 2013; Raha-Chowdhury et al., 2015). To assess this aspect, we extended our investigation of hepcidin expression in microglia and macrophages in control, AD and DS brain samples (*n* = 10 from each group, Table 1) with a particular focus on identifying hepcidin expression in the Iba1 positive microglia. The brain sections from AD, DS and controls were stained with microglial marker (Iba1) and hepcidin. In control brains, Iba1 positive ramified microglia were visible in the cortex and in the blood vessels. There was a limited co-localization with hepcidin in some cells but not with Iba1 positive microglia (Figures 4A–C). In the AD brain, sections from the dentate gyrus (DG), where most of the granule cells were damaged, Iba1 positive activated microglia were present close to the neurons, whereas hepcidin expression was visible only in the damaged granule cells with limited co-localization (Figures 4D–F). These findings indicate that there are different populations of microglia sub-types present in the human brain. To evaluate the effects of inflammatory changes in different microglia, another brain section from AD (MTG) was stained with Iba1 and pro-inflammatory marker IL-1β. Both proteins co-localized in the small plaques and activated microglia (Figure 4G). Similarly, a DS brain section, when stained with Iba1 and hepcidin, Iba1 positive microglia was visible close to blood vessels but there was no co-localization with hepcidin (Figures 4H,I).

As previously reported, the choroid plexus (CP) is damaged in AD brain and as CP is a conduit between the peripheral circulation and central nervous system *via* the cerebrospinal fluid (Raha-Chowdhury et al., 2019). We extended our investigation to CP sections from DS brain, and stained with hepcidin and CD68 (a phagocytotic macrophages/microglia marker). Hepcidin protein was present in the lateral ventricles in a monolayer of epithelial cells of the choroid plexus and CD68 was found in the macrophages close to CP and their co-localization was observed (Figure 4J). These findings suggest that hepcidin might be transported through exosomes or other small vesicles and *via* macrophages to the brain parenchyma. To investigate this phenomenon, another section from AD brain close to the lateral ventricle was stained with CD68 and hepcidin. A large number of CD68 positive peri-vascular macrophages were visible around the lateral wall of the 4th ventricles that co-localized with hepcidin, at the endothelial margin (Figure 4K). Previously, we have shown in the mouse brain that macrophages are present on the pial surface (Raha et al., 2017). We further analyzed a cortical section from DS brain and stained with CD68 and hepcidin. The granular hepcidin protein was visible in the glial cells inside cortical layers I and II and CD68 was visible in the meningeal macrophages (Figure 4L). These data support the notion that soluble and vesicular hepcidin could be transported *via* macrophages and blood vessels into the brain parenchyma.

### IL-6 Could be Involved in Host Defense in AD and DS-Brain

**Figure 5:**
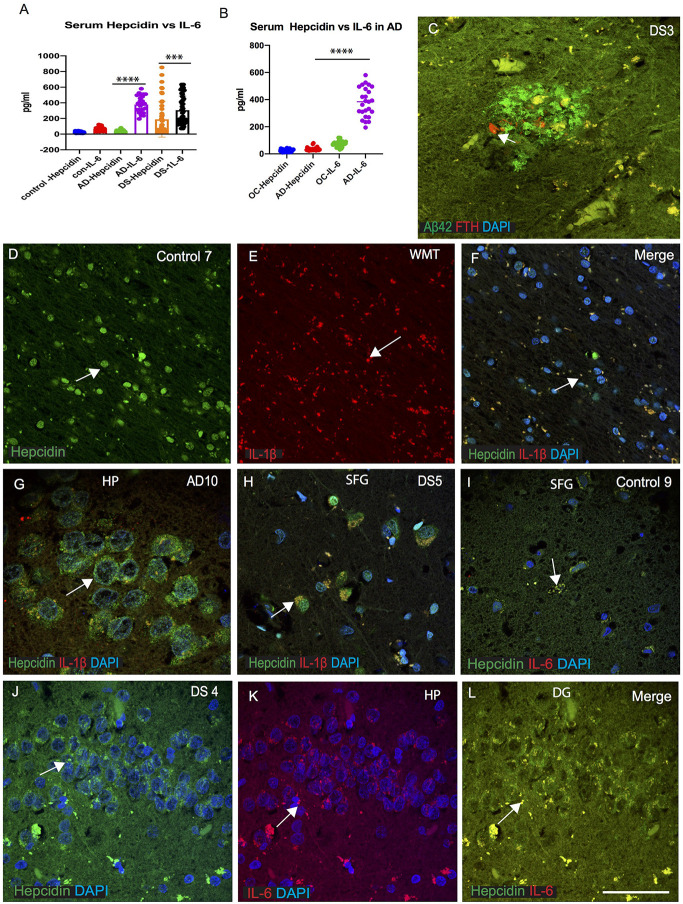
IL-6 could be involved in host defense in AD and DS brain. ELISA based analysis of IL-6 in serum samples showing increased levels in AD and highest level in DS when compared with hepcidin (*p* < 0.0001; **A**). Samples were analyzed from AD and age matched older controls (OC), IL-6 levels were significantly higher in AD serum (**B**; *p* < 0.0001). Ferritin (FTH) was present close to a SP in DS brain analyzed by double immunofluorescence, and did not co-localize with Aβ42 positive plaques **(C)**. Another control brain section from white matter tract (WMT) of corpus callosum (CC), labeled with hepcidin, expression being visible in the WMT (in the oligodendrocytes), whereas IL-1β being present in the microglia with minimal co-localization **(D–F)**. Brain sections from AD (HP, hippocampus) and DS (SFG) when stained with hepcidin and IL-1β, revealed similarly the presence of hepcidin (punctuate, vesicular appearance) in the hippocampal neurons, whereas IL-1β was visible only in the microglia with minimum co-localization **(G,H)**. When control and DS brain sections of HP and SFG were stained for hepcidin and IL-6, there was less IL-6 visible in the control brain **(I)**, whereas there was a higher co-localization seen in the granule cells of hippocampus of DS subjects **(J–L)**. Scale bar: **(C** and **G)** = 20 μm, **(D–F)** = 50 μm, **(H–L)** = 25 μm. ***p < 0.001, ****p < 0.0001.

As we have seen, macrophages play a significant role in enabling iron to enter the plasma compartment. During infection and inflammation, IL-6 and other cytokines aid to increase the synthesis of hepcidin leading to sequestration of iron in the macrophages (Ganz, 2012). The increased levels of hepcidin in the serum described above (in Figure 1C), led us to measure serum IL-6 in AD, DS and controls and compare these results with serum hepcidin (Figures 5A,B). Serum IL-6 levels were significantly increased in AD compared to old-age-matched controls (Figure 1B) and in DS participants (Figure 1A, *p* < 0.0001). In controls, however, serum IL-6 level was lower than AD and DS subjects, suggesting that serum IL-6 could be involved in host defense mechanism in neuroinflammation (Figures 5A,B). We have also shown that expression levels of iron proteins (ferritin, hepcidin) are very different in the brain parenchyma compared to the periphery (in serum; Raha et al., 2013). DS brain sections were stained for Aβ42 and ferritin heavy chain (FTH). Both proteins were found to be co-localized close to the blood vessels, but FTH positive glia was only visible in the SP and did not co-localize with Aβ42 positive cells (Figure 5C). We had previously reported that in the rat brain, hepcidin and FPN expressed in the white matter tract (WMT) of corpus callosum (CC) and was involved in myelination (Raha-Chowdhury et al., 2015). In control brain, hepcidin protein expression was visible in the WMT (in the oligodendrocytes), whereas IL-1β was present in the microglia with minimal co-localization (Figures 5D–F). Brain sections from AD hippocampus (HP) and DS (SFG) when stained for hepcidin and IL-1β, revealed similarly the presence of hepcidin (punctate, vesicular appearance) in the hippocampal neurons, whereas IL-1β was visible only in the microglia with minimum co-localization (Figures 5G,H). Similarly, when control and DS brain sections of HP and SFG were stained for hepcidin and IL-6, there was less IL-6 visible in the control brain (Figure 5I), whereas there was a higher co-localization in the granule cells of HP in DS subjects (Figures 5J–L) suggestive of hepcidin and proinflammatory cytokines (IL-1β and IL-6) being involved in host defense during neuroinflammation.

## Discussion

Alzheimer’s disease, Down’s syndrome and age associated dementia leads to a variety of inflammatory symptoms, and disproportionately endangers people with pre-existing chronic conditions including cardiovascular disease, diabetes mellitus, hypertension, and age-related frailty (Friedland and Haribabu, 2020; Raha et al., 2020; Mukaetova-Ladinska et al., 2021). These populations are not only at a high risk for chronic infection, but also of prevalent SARS-COV-2 infection (Wright et al., 2008). Iron metabolism and anemia may additionally play an important role in multiple organ dysfunction syndrome such as that seen in SARS-COV-2 (Gómez-Pastora et al., 2020; Taneri et al., 2020).

Iron is an essential nutrient for almost all living organisms but when in excess, it is toxic and regulatory mechanisms have evolved to ensure that iron homeostasis is maintained at both the whole-body and cellular levels (Hentze et al., 2010). Ferritin, a highly conserved iron-binding protein and storage for iron, has emerged as a key molecule in the immune system, playing an important role in cellular defense against inflammation (Raha-Chowdhury et al., 1996). Iron is required for synthesis of many key enzymes, including myelin and neurotransmitter production. There are extensive interactions between the brain iron accumulation and the innate immune system in AD and other neurodegenerative diseases (Wright et al., 2008).

Hepcidin has emerged as the key regulatory molecule that controls systemic iron homeostasis in mammals (Krause et al., 2000; Park et al., 2001; Pigeon et al., 2001). Hepcidin is synthesized by the liver and regulates the delivery of iron into the circulation from macrophages, duodenal enterocytes and hepatocytes (Ganz and Nemeth, 2011). Hepcidin achieves this level of control by binding to FPN which is the only known iron exporter expressed by mammalian cells (Nemeth et al., 2004b). On binding with FPN at the cell surface, hepcidin triggers the internalization, ubiquitinoylation and lysosomal degradation of the complex thus limiting cellular iron export (Rivera et al., 2005).

Macrophages also play a key role both in iron homeostasis and in a vast range of biological activities, such as cellular development, scavenging and recycling, tissue repair and host defense (Pollard, 2009). The release of iron from macrophages into plasma, in response to systemic iron requirements, is managed *via* the interaction of hepcidin with its exporter FPN. In humans, macrophages contribute to most of the iron entering the plasma compartment. During infection and inflammation, cytokines, including IL-6, increase the hepcidin synthesis and cause iron sequestration in macrophages (Nemeth et al., 2004a). The resulting decrease in iron availability in tissues can limit the growth and pathogenic impact of invading extracellular microbes, providing an important means of host defense (Ganz, 2012).

The aim of this study was to investigate the expression of iron proteins ferritin and hepcidin, in AD and DS serum and compare these protein levels in brain parenchyma in normal and dementiarelated diseased brain and discuss these findings in relation to the reported hyperferremia in SARS-COV-2 infected patients (Dahan et al., 2020; Gómez-Pastora et al., 2020; Hippchen et al., 2020; Zhou et al., 2020). These findings may explain why people with AD and DS are at a higher risk for SARS-COV-2 infection.

We reported that serum iron and ferritin levels were significantly higher in normal controls compared to AD and DS participants. In contrast, the hepcidin levels were 20 times higher in DS than those detected in AD and controls suggesting the presence of inflammatory changes or dis-erythropoiesis affecting especially the DS subjects.

Using IHC, we analyzed brain iron and other proteins (i.e., ferritin and hepcidin) from AD subjects, and compared with our previously published work on AD brain and transgenic mouse model of AD (APP-PS1; Raha et al., 2013). In the DS brain, abundant Aβ42 positive plaques were present in the neocortex, similar to those seen in AD brain, and additionally when stained with Congo red showed the existence of fibrillary birefringence in SP, and Perls stains in glial cells, indicating the presence of iron in the SP of DS brain as previously reported (Morris et al., 1992; Howie et al., 2008). We have noted amyloid deposition within blood vessels signifying presence of cerebral amyloid angiopathy which is widely reported in almost all DS brains. We noted several meningeal vessels were positive for Aβ42 (Figure 1I). In Figures 2D–F, DS brain section stained with Aβ42 and analyzed by confocal microscope, showed amyloid expression in the blood vessel walls too. Similarly, in Figure 3C (hepcidin in the blood vessels) and 3F (FTL within blood vessel walls), and Figure 1I showed Aβ42 deposit in the blood vessels.

All DS subjects had aneuploidy, i.e., carried an extra copy of chromosomes 21, where APP gene is encoded. DS subjects do produce a much higher amount of amyloid β protein due to carrying an extra copy of APP gene, that subsequently produce excess amount of Aβ affecting its clearance from the blood vessels (Raha et al., 2013; Raha-Chowdhury et al., 2018). Our IHC analysis indicated that amyloid plaques were carrying ferritin in the core of SP, and consequently the iron accumulation could lead to increase in oxidative stress in DS subjects.

In the neocortex of AD and DS subjects, Aβ42 positive SPs were visible close to the blood vessels, while hepcidin predominated in the GFAP positive astrocytes in granular form and in the endothelial cells of blood vessels. Similarly, in AD brain, astrocytes were present surrounding the blood vessels and co-localized with FTL. These findings support the notion that astrocytes have a task in iron transport in the brain parenchyma. FTL and hepcidin may enter the brain from the periphery *via* blood vessels or could even export proteins from the brain parenchyma to the peripheral circulation.

Hepcidin and ferritin are also involved in microglial activity and the amyloid clearance process (Raha et al., 2013; Raha-Chowdhury et al., 2015). In AD brain section, Iba1 (a microglial marker) positive activated microglia were present close to the SP, whereas hepcidin expression was visible in the damaged granule cells of dentate gyrus (DG) but did not co-localize with Iba1. These findings indicate that there are different population of microglial sub-types present in the human brain and some of the Iba1 positive activated microglia are involved in pruning and clearance of dead cells, and co-localize with pro-inflammatory marker IL-1β but not with hepcidin. Our observations support previously reported notion that hepcidin also has a special role in repair and regeneration of glial cells and neural plasticity (Forostyak et al., 2020).

Previously, we have reported that the choroid plexus (CP) is damaged in AD brain, and CP is a conduit between the peripheral circulation and the central nervous system *via* the cerebrospinal fluid (Raha-Chowdhury et al., 2019). Our findings of hepcidin (present in a monolayer of cuboidal epithelial cells of the CP) and CD68 (present in the macrophages close to CP) and their colocalization, as well as their ventricular and cortical localization, support the notion that soluble and vesicular hepcidin could be transported both *via* different population of macrophages (CP, perivascular and meningeal macrophages) and *via* the blood vessels in to the brain parenchyma (Kalaria et al., 1996).

The hyperinflammatory response induced by SARS-CoV-2 through peripheral macrophages is a major cause of disease severity (Cohen et al., 2010). This population of innate immune cells sense and respond to microbial threats by producing inflammatory molecules that eliminate pathogens and promote tissue repair. A dysregulated macrophage response, however, can be damaging to the host, as seen in the macrophage activation syndrome induced by severe infections, including SARS-CoV-2 (Otsuka and Seino, 2020).

Microglia in the vicinity of the SP and NFT in AD/DS become activated and release a cocktail of inflammatory cytokines and chemokines (Akiyama et al., 2000). The persistence of infection and its impact on macrophages, microglia and astrocytes in the CNS are particularly important since a neurotropic virus can activate glial cells and induce a pro-inflammatory state (Kanberg et al., 2020).

Inflammation in AD and DS could further be a reason for the increase in hepcidin levels as noticed in the systemic environment. Besides the iron levels present in the circulation, inflammation is yet another factor regulating hepcidin transcription in the hepatocytes. Inflammatory cytokines, mainly in the form of IL-6, are released during inflammation and induce hepcidin expression in the hepatocytes (Pietrangelo et al., 2007; Eikelenboom et al., 2012a). IL-6, a major highly inducible pro-inflammatory cytokine, is secreted by many different cell types including monocytes, lymphocytes, fibroblasts, and endothelial cells (Zegeye et al., 2018).

Not surprisingly, we confirmed increased serum IL-6 levels in AD and DS subjects, while controls had IL-6 levels lower than that of ferritin suggesting that serum IL-6 could be involved in host defense in AD and DS. An increased serum level of IL-6 is linked to severity (Ulhaq and Soraya, 2020a) and increased fatality in SARS-COV-2 (Chan et al., 2020), as well as respiratory dysfunction (Ulhaq and Soraya, 2020b). Similar findings have now been reported using profiling plasma proteomics that not only identified IL-6 to be among the most perturbed proteins in SARS-COV-2 patients but also confirmed it as an indicator of disease severity. In addition, the rapid replication of SARS-CoV-2 triggers elevated IL-6 production that can lead to heightened respiratory distress. Therefore, IL-6 stands as a possible common biomarker for AD/DS and SARS-COV-2 (Hüll et al., 1996).

Levels of IL-1 have also been reported to have increased in AD and DS patients (Griffin et al., 1989). IL-1 has been noticed to be significantly higher in the SARS-COV-2 patients too during the disease onset as well as throughout the duration of disease progression (Cavalli et al., 2020).

Apolipoprotein E is the main carrier of cholesterol in the central nervous system (CNS) and an important constituent of very low–density lipoproteins (VLDL). Among its three alleles (ε2, ε3, and ε4), the individuals carrying the ε4 allele are at a higher risk of developing AD since the ApoE ε4/ε4 genotype increases fibrinogenesis in the brain of Alzheimer’s disease patients (Hultman et al., 2013; Raha-Chowdhury et al., 2018). In our DS cohort, more than 25% subjects were carrying heterozygous ApoE ɛ4 allele and interestingly all such subjects had higher serum IL-6 levels (average 465.85 pg/ml) compared to the DS subjects carrying ApoE ε2/ε3 allele (average 129.93 pg/ml; Henderson et al., manuscript is in progress). Recently, ApoE ε4 has been regarded as a marker that could be indicative of increasing severity risk in SARS-COV-2 (Kuo et al., 2020). Our findings of higher inflammatory markers in ApoE ε4 carriers provide further evidence to suggest that AD and DS patients carrying the ApoE4 allele are at a heightened risk of developing SARS-COV-2 infection.

Older people are at a higher risk of falling victim to both AD dementia and SARS-COV-2 infection. Although DS subjects are usually younger than AD subjects, they suffer from accelerated ageing and neuroinflammation that itself are additional risk factors for SARS-COV-2 infection. Heightened production of reactive oxygen species (ROS), iron accumulation, exacerbated production of amyloidβ, its aggregation and consequent neurodegeneration, perturbed lifestyle modification have all been implicated in the pathogenesis of AD, and all such comorbidities also place them at a higher risk for SARS-COV-2 infection.

## Conclusion

AD and DS subjects are likely to be both at a higher risk for SARS-COV-2 infection due to dis-balance in iron homeostasis and failure of amyloid protein clearance leading to neuro-inflammation. Macrophages plays a key role in iron homeostasis, regulating passage of iron proteins between the brain parenchyma and the peripheral circulation. Macrophages are a population of innate immune cells that sense and respond to microbial threats by producing inflammatory molecules that eliminate pathogens and promote tissue repair. DS, AD and SARS-COV-2 share common links with respect to iron proteins, hepcidin, ferritin and pro-inflammatory markers such as interleukin-1 (IL-1β), IL-6, and ApoE ɛ4 allele. Hepcidin, ferritin and IL-6 participate significantly towards host defense mechanism associated with neuroinflammation. Such mechanisms could well be implicated in SARS-CoV-2 infection and objective evaluation of perturbed iron homeostasis may be indicative of how severely someone may be infected by SARS-CoV-2.

## Data Availability Statement

The original contributions presented in the study are included in the article, further inquiries can be directed to the corresponding author/s.

## Ethics Statement

The studies involving human participants were reviewed and approved by Ethics plus research and development (R&D) and approvals were granted by the National Research Ethics Committee of the East of England—Norfolk and Cambridgeshire and Peterborough NHS Foundation Trust, respectively. Cambridge Health Authorities Joint Ethics Committee granted ethical approval for the use of human brain tissue and serum samples (Project ref no.: REC:15/WM/0379). Written consents were obtained from controls, adults DS participants and subjects with Alzheimer’s disease (AD) with the capacity to consent. Verbal assent was obtained from participants with AD and DS, who lacking capacity to provide written assent, and this was provided instead by an appointed consolutee, in accordance with the Mental Capacity Act of the UK (2005). The patients/participants provided their written informed consent to participate in this study.

## Author Contributions

AR and JH performed biochemical analysis. SC critically evaluated the results and edited the manuscript. AR and SG performed tissue analysis and confocal microscopy. SZ, AH and RF evaluated clinical findings. EM-L evaluated clinical samples and edited the manuscript. KA selected the brain samples and preliminary assessment of disease status. RR-C and AR contributed to the hypothesis development, analyzed the results and edited the manuscript. RR-C performed study design, supervised the project, critically evaluated the results and wrote the manuscript. All authors contributed to the article and approved the submitted version.

## Conflict of Interest

The authors declare that the research was conducted in the absence of any commercial or financial relationships that could be construed as a potential conflict of interest.
